# Exenatide with Metformin Ameliorated Visceral Adiposity and Insulin Resistance

**DOI:** 10.1155/2018/4019248

**Published:** 2018-02-06

**Authors:** Xuan Du, Wen Lu, Zijun Lu, Xinyu Shao, Chunhong Hu, Bimin Shi

**Affiliations:** ^1^Department of Endocrinology and Metabolism, The First Affiliated Hospital of Soochow University, Suzhou, Jiangsu, China; ^2^Department of Radiology, The First Affiliated Hospital of Soochow University, Suzhou, Jiangsu, China

## Abstract

**Background:**

To study the effectiveness of exenatide with metformin and sequential treatment with exenatide and glargine added to metformin and their influence on insulin sensitivity and adipose distribution.

**Methods:**

20 newly diagnosed obese type 2 diabetic patients were enrolled, and 2-month washout treatment of metformin, 6-month exenatide treatment, and 6-month glargine treatment were administrated sequentially accompanied with previous metformin. Glucolipid metabolic parameters were compared among groups. Adipose distribution was quantified with computerized tomography according to anatomy, dividing into visceral adipose tissue (VAT) and subcutaneous adipose tissue (SAT), adding up to total adipose tissue (TAT).

**Results:**

The 6-month exenatide treatment dramatically ameliorated the glucose and lipid profile, improved insulin sensitivity, and mainly decreased VAT and also the ratio of VAT/SAT (RVS). The following 6-month glargine treatment increased VAT. The whole 12-month sequential treatment with exenatide and glargine added to metformin basically improved the insulin sensitivity and glucolipid control though VAT rebounded at the end, however without deteriorating the other parameters.

**Conclusion:**

Exenatide is an ideal treatment for obese type 2 diabetic patients in the aspect of adipose tissue distribution. Sequential treatment of exenatide and glargine could be an alternative for low-income patients who cannot afford GLP-1 agonist for long time. This trial is registered with ChiCTR-OOC-17013679.

## 1. Introduction

Obesity is epidemic throughout the world. Obesity, especially visceral obesity, could increase the risk of type 2 diabetes, hypertension, dyslipidemia, and atherosclerosis, suggesting that obese patients with type 2 diabetes are at high risk for cardiovascular diseases [1]. Chinese people, though traditionally considered relatively leaner when evaluated with the body mass index (BMI), have a high prevalence of visceral obesity, making them susceptible to type 2 diabetes mellitus [2]. Which one is the origin that leads to the metabolic mess, obesity or insulin resistance? Recent understanding on visceral adiposity could partly explain the dilemma between obesity and insulin resistance [3]. Thereafter, treatments which can decrease visceral adipose tissue were considered better choices.

Exenatide is a synthetic derivative of exendin-4, a peptide found in the saliva of the Gila monster [4]. As a glucagon-like peptide-1 agonist (GLP-1 agonist), exenatide was approved in 2005 for the treatment of type 2 diabetes mellitus [4]. It was reported that exenatide could stimulate the secretion of insulin and reduce body weight in different ethnic groups [4, 5]. It is also widely believed that the GLP-1 agonist could influence the energy intake-output balance by suppressing the appetite and changing the network of insulin-glucagon, therefore affecting the adipose tissue distribution [6]. In the study described below, we presented an uncontrolled real-world evidence (RWE) study of a possible on-target treatment with a glucagon-like peptide-1 agonist, observing its effects on visceral adiposity and insulin resistance.

## 2. Materials and Methods

A total of 20 newly diagnosed obese type 2 diabetic patients (age 51.0 ± 10.5 years) were enrolled, including 9 males and 11 females. The inclusion criteria were as follows: (1) type 2 diabetes mellitus according to the criteria of the WHO of 1999, (2) Chinese adult aged 30~65, (3) BMI equal to or greater than 28 kg/m^2^, and (4) HbA1c levels between 7 and 11% under 2-month treatment of metformin (daily dosage not less than 1500 mg). The exclusion criteria were as follows: (1) type 1 diabetes mellitus, (2) allergic history to incretin mimetics, (3) interstitial pneumonia, (4) severe renal or liver disease, (5) malignant tumors, and (6) history of acute coronary syndrome or stroke or acute pancreatitis within 3 months prior to enrollment.

The glycated hemoglobin (HbA1c) levels were measured with the HPLC method on a Bio-Rad Variant II glycosylated hemoglobin analyzer (San Diego, CA, USA). Fasting insulin (FINS) and C-peptide (CP) were detected with the radioimmunoassay (RIA). Fasting/postprandial blood glucose and blood lipid profile including total cholesterol (TC), total triglyceride (TG), high-density lipoprotein-cholesterol (HDL-c), and low-density lipoprotein-cholesterol (LDL-c) were detected with the ADVIA 2400 Chemistry System (Siemens, Munich, Germany) as routine clinical tests in the hospital.

**Figure 1:**
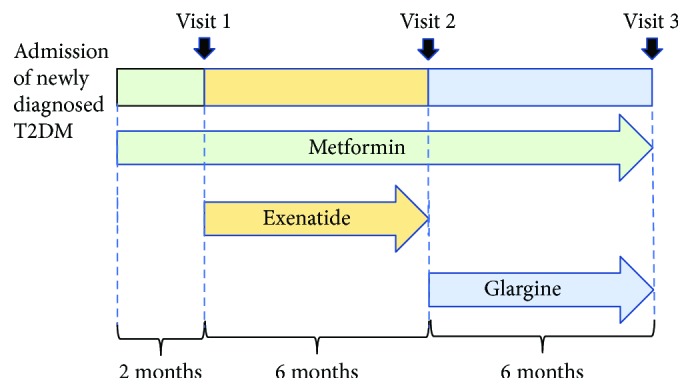
The procedure of treatment and observation.

The design and procedure of the study were shown in Figure 1. The study started with 2-month washout treatment of metformin (daily dosage not less than 1500 mg). Thereafter, exenatide injection was added, starting with 5 mcg injection subcutaneously twice daily for one month, and then followed by 10 mcg injection subcutaneously twice daily for the next five months. Body weight, body mass index (BMI), waist circumference, hip circumference, and waist-to-hip ratio (WHR) were recorded and compared. When the 6-month exenatide treatment ended, glargine was used to combine with previous metformin treatment, and the treatment was followed up for another 6 months. The initial dosage was started with 0.2 unit/kg and then modified according to the fasting blood glucose, adding two units in every modification when fasting glucose was higher than 7.0 mmol/L. 15 patients came back to evaluate the long-term effect of the sequential treatment. 3 patients chose to continue exenatide treatment and refused to use glargine, while 2 patients quit for inconvenience.

Data from the 20 patients at visit 1 and visit 2 and 15 patients at visit 3 were statistically compared. The insulin resistance index was calculated by homeostasis model assessment of insulin resistance (HOMA-IR) as (fasting insulin (mU/L) × fasting glucose (mmol/L))/22.5. Beta-cell secretion function was calculated by homeostasis model assessment of *β* as (20 × fasting insulin (mU/L))/(fasting glucose (mmol/L) − 3.5).

Computerized tomography (CT) was performed with a Somatom Emotion 16 CT scanner (Siemens, Munich, Germany) by one designated specialist. Patients were scanned above the level of S1 in a supine position. Briefly, 25 contiguous 5 mm thick slices (120 kV; 400 mA; gantry rotation time, 500 ms; table feed, 3 : 1) were acquired covering 125 mm above the level of S1. Images were reconstructed with Volume Viewer 3.1 software on an AW Workstation (General Electric, Milwaukee, WI, USA) [7]. The muscular wall which separates the two adipose tissue compartments was manually traced. Visceral adipose tissue volumes (VAT volumes), subcutaneous adipose tissue volumes (SAT volumes), and total adipose tissue volumes (TAT volumes) were measured by drawing contours of adipose tissue, automatically determined, and were calculated in cm^3^. And then the VAT/SAT ratio was calculated.

All statistical analyses were carried out using the Statistical Package for Social Sciences (SPSS version 12.0 for Windows, Chicago, IL, USA). Results are expressed as mean ± standard deviation (SD). The mean values of variables are studied by analysis of variance (ANOVA). Pearson correlation was used to analyze the relation between abdominal fat and other clinical parameters. The level of significance was accepted as *p* < 0.05.

## 3. Results

### 3.1. Baseline Measurements

Data from the 20 patients showed that the baseline glycated hemoglobin (HbA1c) was 9.745 ± 1.50%, while the baseline fasting blood glucose was 13.07 ± 2.36 mmol/L. BMI was 29.77 ± 1.49 kg/m^2^. Age is 51 ± 10.5 years.

To analyze the parameters influencing the adipose distribution pattern before any medical therapy, Pearson correlation was performed. Results indicate that TAT was positively correlated with BMI, waist circumference, LDL-c, HbA1c, FINS, CP, and HOMA-IR (*r* = 0.813, 0.822, 0.665, 0.464, 0.484, 0.643, and 0.518, resp., *p* < 0.05). VAT was positively correlated with BMI, waist circumference, TG, LDL-c, HbA1c, FINS, CP, and HOMA-IR (*r* = 0.646, 0.625, 0.635, 0.549, 0.609, 0.569, 0.667, and 0.532, resp., *p* < 0.05) and negatively correlated with HDL-c (*r* = −0.529, *p* < 0.05). In comparison, SAT was only positively correlated with BMI and waist circumference (*r* = 0.497 and 0.644, *p* < 0.05). The ratio of VAT/SAT was positively correlated with TG and FINS (*r* = 0.641 and 0.469, *p* < 0.05).

### 3.2. Comparison between Pre- and Postexenatide Treatment

**Figure 2:**
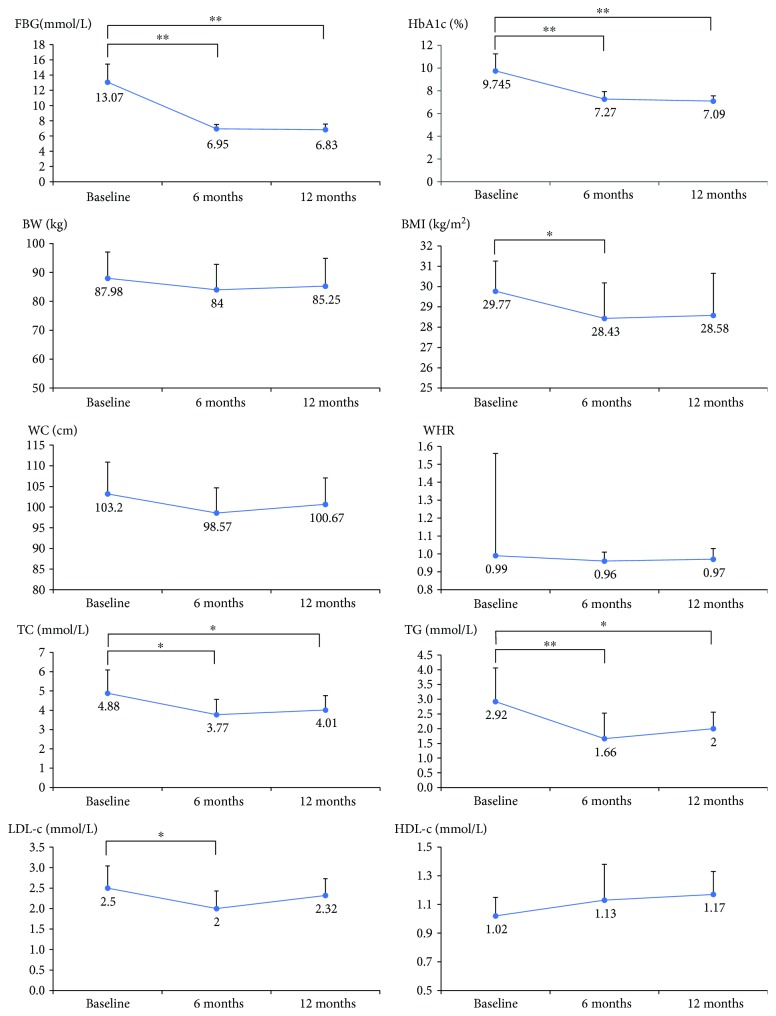
The parameters of glucose and lipid metabolism among the pre- and post-6-month exenatide treatment and the following 6-month glargine treatment. Data are expressed as mean with standard deviation (SD) (^∗^*p* value < 0.05; ^∗∗^*p* value < 0.01). FBG: fasting blood glucose; HbA1c: hemoglobin A1c; BW: body weight; BMI: body mass index; WC: waist circumference; WHR: waist-to-hip ratio; TC: serum total cholesterol; TG: serum total triglyceride; LDL-c: low-density lipoprotein-cholesterol; HDL-c: high-density lipoprotein-cholesterol.

**Figure 3:**
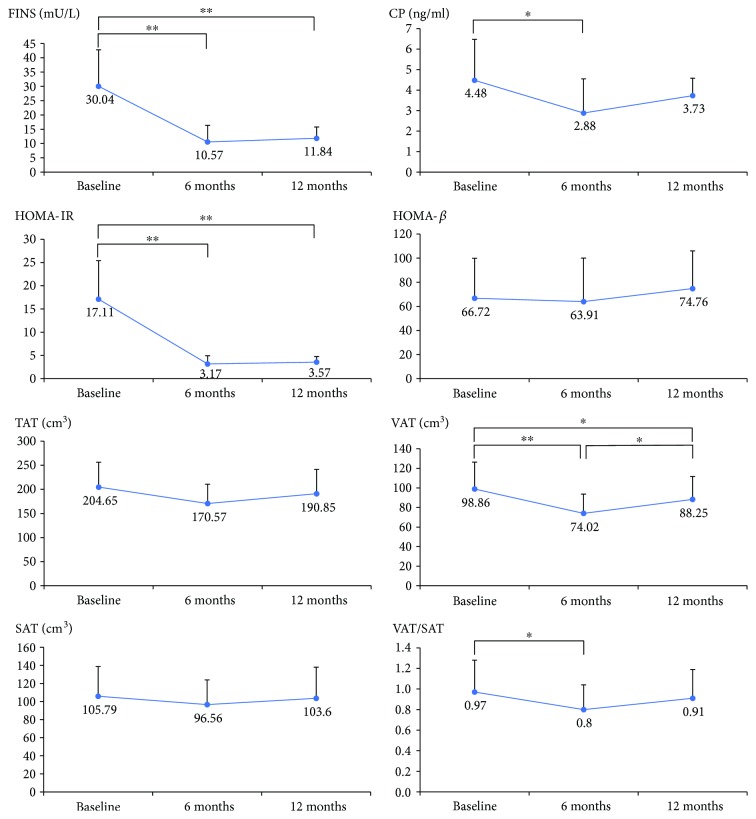
The parameters of insulin sensitivity and adipose distribution among the pre- and post-6-month exenatide treatment and the following 6-month glargine treatment. Data are expressed as mean with standard deviation (SD) (^∗^*p* value < 0.05; ^∗∗^*p* value < 0.01). FINS: fasting serum insulin level; CP: fasting serum C-peptide level; HOMA-IR: homeostatic model assessment of insulin resistance; HOMA-*β*: homeostatic model assessment of beta-cell function; TAT: total adipose tissue volumes; VAT: visceral adipose tissue volumes; SAT: subcutaneous adipose tissue volumes; VAT/SAT: ratio of visceral adipose tissue volumes (VAT) over subcutaneous adipose tissue volumes (SAT).

As shown in Figures 2 and 3, after the six-month exenatide treatment, FBG, HbA1c, BMI, fasting insulin, fasting C-peptide, total cholesterol, total triglyceride, LDL-c, and HOMA-IR of the patients decreased compared with those before the treatment (*p* < 0.05).

As for the change of abdominal adipose tissue volume after the six-month exenatide treatment, VAT volumes decreased significantly, while SAT volumes and TAT volumes decreased slightly, without statistical significance. The ratio of VAT/SAT after the treatment was lower than that before. These results indicated that exenatide mainly functions on the visceral adiposity.

### 3.3. Glargine Dosage following the Exenatide Treatment

After the 6-month exenatide treatment ended, glargine was used to take the place of exenatide. Glargine was started with 0.2 unit/kg and then modified according to the fasting blood glucose, adding two units every time when fasting glucose was higher than 7.0 mmol/L. At the end of the 6-month treatment, the final daily dosage of glargine was 23.01 ± 4.94 units or 0.27 ± 0.04 unit/kg, similar to that of previous reports [8].

### 3.4. Comparison among Baseline, after Exenatide and Glargine Treatment

Patients were called back to repeat the tests mentioned above in the three major visits, that is, at the end of the single metformin washout period, exenatide plus metformin period, and exenatide plus metformin period. At the end of the whole 12-month sequential treatment, though most people rebound a little in body weight and adipose tissue amount, nobody regains their body weight completely. Their blood glucose levels kept stable compared with those of exenatide treatment (FBG 6.95 ± 0.56 mmol/L versus 6.83 ± 0.75 mmol/L and HbA1c 7.27 ± 0.66% versus 7.09 ± 0.46%, *p* value > 0.05). The glucose control level with exenatide and the following glargine treatment seemed to have no significant difference.

At the end of the exenatide treatment, FINS and HOMA-IR decreased dramatically to around 1/3 and 1/5 those of baseline level, with HOMA-*β* roughly unchanged. And after the 6-month treatment of glargine, the level of FINS and HOMA-IR also kept relatively stable. The lowering of blood glucose and the simultaneous decrease in insulin secretion might explain the alleviation of insulin resistance. And the whole 12-month sequential therapy with exenatide and glargine alleviated the insulin resistance and lipid profile with slight influence on BMI after exenatide treatment, without much changes on body weight, waist circumference, or waist/hip ratio. As for the adipose distribution, the most obvious change took place on VAT. As seen in Figure 3, exenatide decreased the visceral adipose tissue volume, while glargine increased it. But the effect of exenatide overwhelmed that of glargine, for VAT at the end of 6-month injection of glargine was still smaller than that at baseline.

It seems that the 6-month exenatide treatment had beneficial influence longer than expected, even when the exenatide treatment has already ceased for 6 months.

## 4. Discussion

Anatomically, abdominal adipose tissue can be divided into visceral and subcutaneous adipose tissues, which are different both in position and function [9]. Abnormal accumulation of visceral adipose tissue is considered a risk factor for type 2 diabetes mellitus and cardiovascular diseases. Visceral adipose tissue is characterized by high activity of lipogenesis and lipodieresis, producing a large amount of free fatty acid (FFA) pouring into the liver, causing insulin resistance and exacerbating diabetes [10]. Distribution of visceral and subcutaneous adipose tissue was basically the question where the lipid goes. In our previous understandings, subcutaneous adipose tissue is considered a *good fat*, while the visceral counterparts a *bad fat* [11].

Glucagon-like peptide-1 (GLP-1) receptor agonists, used as glucose-lowering drugs, can also reduce body weight by decreasing food intake and reversing hepatic steatosis [12]. According to the report of Inoue et al., a short-term treatment of liraglutide effectively reduced visceral fat adiposity, which was estimated with waist circumference, waist/hip ratio, and estimated visceral fat area measured by abdominal bioelectrical impedance analysis (BIA) [13]. In order to measure more accurately and also to expand observation to exenatide, we used the CT scan and quantification method. And because most of the lipid stored in adipose tissue is in the form of triglyceride, serum TG level is understandably closely related to the distribution of fat, as shown in our present study, which indicated that VAT and the ratio of VAT/SAT both positively correlated with serum TG level. It seems that the elevation of serum TG is not related to subcutaneous adipose tissue and the unspecific TAT, which equals to SAT plus VAT. The results showed that the visceral adipose tissue had more close relation with human dyslipidemia than its subcutaneous counterpart did.

By comparing exenatide with glargine in their 6-month treatment combined with metformin, they had comparable glycemic and lipid control and their fasting glucose, HbA1c, TG, TC, FINS, and HOMA-IR were similarly lower than those of the pretreatment, making exenatide and glargine both reasonable methods for newly diagnosed obese type 2 diabetic patients, from the aspect of glucose/lipid control and insulin resistance. But from the aspect of adipose tissue distribution and visceral obesity, exenatide and glargine are different in the fact that exenatide decreased the visceral adiposity while glargine increased it (Figure 3). In consideration of relatively short durations, we had not observed whether VAT would reverse to or even higher than that of baseline level if glargine treatment was prolonged to a longer period, for example, one year or more. At least at the end of the second 6-month period, VAT volumes were still smaller than those before the injections. The sequential treatment of exenatide followed by glargine still improved the distribution of *good* and *bad* adipose tissue. It was reported that exenatide could improve *β*-cell function up to 3 years in patients with type 2 diabetes [14]. However, in our study, HOMA-*β* which represents the insulin secretion function had no significant change during the whole periods of the treatments. Exenatide and glargine seemed to affect more insulin sensitivity rather than insulin secretion. The change of sensitivity might be related partly to glucose control or adipose distribution.

For the low-income level, most Chinese patients cannot afford GLP-1 agonists for long term in consideration of their high price and no coverage by local medical insurance. Consequently, many patients had to switch to other remedies after several months of GLP-1 agonist treatment. According to our own unpublished survey in our hospital during the past years, most people could only stick to GLP-1 agonists for 3 to 12 months; then, they had to change to insulin, sulfonylureas, metformin, or DPP4 inhibitors, mostly for the economic reason. Therefore, our study aimed to simulate what is happening every day in our real world. In our observation, there were three patients that chose to pay for exenatide because it gave them confidence with fine blood glucose and body weight control. But because of their small number and higher-income level, the possible difference on their willingness and compliance to therapy might lead to unavoidable statistical confounding; therefore, we did not put these three patients into the comparison and statistics. But VAT volumes of these three patients also reached a plateau, making our sequential treatment with exenatide and glargine a cheaper alternative of long-term GLP-1 agonist, at least within 12 months. More clinical trials may be needed to confirm more affordable treatments.
